# A phase 2, open-label, multi-center study of amuvatinib in combination with platinum etoposide chemotherapy in platinum-refractory small cell lung cancer patients

**DOI:** 10.18632/oncotarget.19888

**Published:** 2017-08-03

**Authors:** Lauren Averett Byers, Leora Horn, Jitendra Ghandi, Goetz Kloecker, Taofeek Owonikoko, Saiama Naheed Waqar, Maciej Krzakowski, Robert J. Cardnell, Junya Fujimoto, Pietro Taverna, Mohammad Azab, David Ross Camidge

**Affiliations:** ^1^ University of Texas, M.D. Anderson Cancer Center, Houston, TX, USA; ^2^ Vanderbilt-Ingram Cancer Center, Vanderbilt University Medical Center, Nashville, TN, USA; ^3^ Associates in Oncology and Hematology, Chattanooga, TN, USA; ^4^ James Graham Brown Cancer Center, University of Louisville, Louisville, KY, USA; ^5^ Winship Cancer Institute, Emory University, Atlanta, GA, USA; ^6^ Siteman Cancer Center, Washington University School of Medicine, St Louis, MO, USA; ^7^ Centrum Onkologii-Instytut Im. M. Skłodowskiej-Curie w Warszawie, Warszawa, Poland; ^8^ Astex Pharmaceuticals, Inc. Pleasanton, CA, USA; ^9^ Anschutz Cancer Pavilion, University of Colorado Cancer Center, Aurora, CO, USA

**Keywords:** amuvatinib, MP-470, platinum-refractory, etoposide, SCLC (small cell lung cancer)

## Abstract

**Background:**

Amuvatinib (MP-470) is a multi-targeted kinase inhibitor with potent activity against c-Kit, synergistic with DNA-damaging agents. We evaluated amuvatinib in combination with platinum-etoposide (EP) chemotherapy by objective response rate, survival, and tolerability in platinum-refractory small cell lung cancer (SCLC) patients.

**Methods:**

This study used a Simon 2-stage design requiring ≥3 centrally confirmed responses in the first 21 subjects. Subjects received EP with 300 mg amuvatinib orally three times daily in cycles of 21 days. A three-day amuvatinib run-in period before EP occurred in Cycle 1. Subjects received the same EP chemotherapy regimen given prior to progression/relapse.

**Results:**

Among 23 subjects treated, we observed four PRs (17.4%) per RECIST 1.1, only two of which were centrally confirmed (8.7%, response duration 119, 151 days). Three subjects (13%) had confirmed stable disease. c-Kit H-score was ≥100 in two subjects whose respective durations of disease control were 151 and 256 days.

**Conclusions:**

The addition of amuvatinib to EP chemotherapy in unselected, platinum-refractory SCLC did not meet the primary endpoint of ≥3 confirmed responses in stage 1. However, high c-Kit expression in two subjects with durable disease control suggests the potential for further study of amuvatinib in SCLC patients with high c-Kit expression.

## INTRODUCTION

Small cell lung cancer (SCLC) accounts for 13% of lung cancers diagnosed in the United States and is characterized by aggressive behavior with a high growth fraction and swift development of metastasis [1, 2]. Approximately 60%-70% of patients present with extensive-stage SCLC and inevitably relapse despite initial response to chemotherapy and radiation. As a result, the median survival rate for these patients is only 8-13 months [3, 4]. Early diagnosis of SCLC and effective treatment remain significant challenges in the management of these patients.

In patients with extensive-stage disease, treatment with platinum-etoposide (EP) (cisplatin-etoposide or carboplatin-etoposide) is an established and widely used first-line therapy [5, 4]. In those who experience a complete response (CR), partial response (PR), or who have stable disease (SD), treatment is usually discontinued after 4-6 cycles of chemotherapy. Extended treatment regimens increase toxicity and fail to prolong overall survival [5, 4]. Platinum-refractory patients (i.e. patients who progress while on EP or within 3 months of completing first-line EP) have a guarded prognosis and are typically refractory to additional chemotherapy. For example, topotecan—the only FDA-approved chemotherapy for the second-line treatment of SCLC—has shown extremely limited activity in platinum-refractory SCLC, with an overall response rate of 6.4% [6]. Therefore, patients with platinum-refractory have limited options.

Amuvatinib (MP-470) is a multi-targeted tyrosine kinase inhibitor with potent activity against several validated cancer targets, including mutant forms of c-Kit and platelet-derived growth factor receptor alpha [7], and has been shown to have synergistic activity with DNA-damaging agents, including radiation and topoisomerase inhibitors [8]. The mechanism of action of amuvatinib is postulated to be DNA repair inhibition mediated by down regulation of RAD51 expression [8]. RAD51 is a key protein in the repair pathway for DNA double-strand breaks; it has been shown to be critical to the homologous recombination repair process in cells and can be employed as a mechanism of resistance to DNA-damaging agents in tumor cells. RAD51 down regulation after amuvatinib treatment was associated with reduced ribosomal protein S6 phosphorylation and inhibition of global translation [9]. Hansen et al [10] have shown that etoposide resistance in SCLC cells is positively correlated with RAD51 levels and have suggested that RAD51 is a potential target to improve etoposide efficacy in treatment of SCLC. Previous preclinical studies have shown that amuvatinib in combination with etoposide has a synergistic effect on human SCLC cell lines *in vitro* and SCLC xenografts *in vivo* [11].

Phase 1 clinical studies have shown single-agent amuvatinib to be safe and well tolerated [12, 13], with amuvatinib exposures significantly improved when lipid-suspension capsules rather than dry-powder capsules are administered [12]. In addition, Mita et al [14] showed preliminary evidence of anti-tumor activity and durable SD (up to 708 days) with amuvatinib administered at varying dose levels in combination with standard chemotherapy agents. This included a SCLC patient who had a partial response to amuvatinib in combination with carboplatin-etoposide following multiple prior lines of chemotherapy.

In this ESCAPE study (Tr***E***atment of ***S***mall ***C***ell Lung Cancer with ***A***muvatinib in Combination with ***P***latinum ***E***toposide), we sought to assess the effect of amuvatinib in combination with EP on the objective response rate in subjects with extensive-stage or limited-stage SCLC who had not responded to standard treatment with EP or who had relapsed within 90 days of EP treatment.

## RESULTS

### Enrollment and subject disposition

The study enrolled 24 subjects between September 2011 and May 2012. Enrollment was concluded after Stage 1, as independent assessment showed that among the first 21 subjects, fewer than 3 had a confirmed response.

Of the 24 subjects enrolled, 23 (96%) received amuvatinib + EP and were included in the analysis (one subject was not treated because of abnormally high magnesium levels). Eleven (46%) were withdrawn from the study due to progressive disease, 4 (17%) withdrew due to an adverse event (AE), 3 (13%) withdrew consent, 3 (13%) withdrew due to death, and 3 (13%) withdrew due to physician decision.

### Demographics and baseline characteristics

Table 1 shows subject demographics (*N* = 23). Mean age was 62 years (range, 44-80 years), and 87% were Caucasian. Twenty-two subjects (96%) had extensive-stage disease, 1 (4%) had limited-stage disease, and the number of prior anticancer regimens ranged from one to three. The best response to prior therapy was partial response in 7 subjects (30%) and stable disease in 11 (48%). Two subjects had disease progression after prior therapy with carboplatin/etoposide and had a best response of progressive disease in this study. The most frequently given prior chemotherapy was carboplatin/etoposide (16/23 subjects, or 70%) (Table 2); six subjects received cisplatin/etoposide and one received carboplatin/etoposide for cycle 1 and cisplatin/etoposide for cycles 2, 3, and 4. Subjects began amuvatinib treatment with the same dose and schedule of their last EP chemotherapy regimen in which they progressed or relapsed (Table 3).

**Table 1:** Demographic and baseline characteristics (N = 23)

Characteristic		Number (percent)
**Age (yr)**	Mean	62
	Range	44-80
**Sex**	Male	11 (48)
	Female	12 (52)
**Race**	White	20 (87)
	Black or African American	3 (13)
**ECOG performance status**	0	5 (22)
	1	13 (57)
	2	5 (22)
**Study disease at entry**	Disease progression during EP chemotherapy	9 (39)
	Relapse ≤90 days from previous EP treatment	11 (48)
	SD as best response after at least 2 cycles of EP chemotherapy	3 (13)
**Disease stage**	Extensive-stage	22 (96)
	Limited-stage	1 (4)
**No. of prior anticancer regimens**	Median	1
	Minimum	1
	Maximum	3
**Best response to prior therapy**	Partial response	7 (30)
	Stable disease	11 (48)
	Progressive disease	2 (9)
	Unknown	3 (13)

**Table 2:** Prior chemotherapy regimens by subject

Subject	Total cycles (n)	Agents	Total actual dose (mg)
001	6	Carboplatin Etoposide	3746 2964
002	2	Carboplatin Etoposide	980 966
003	1	Carboplatin Etoposide	652 201
004	4	Carboplatin Etoposide	1819 2283
005	2	Carboplatin Etoposide	1125 1215
006	2	Carboplatin Etoposide	1206 1140
007	1	Carboplatin Etoposide	640 477
008	9	Carboplatin Etoposide	5760 5000
009	6	Carboplatin Etoposide	3800 3222
010	2	Cisplatin Etoposide	304.5 960
011	2	Carboplatin Etoposide	788 165
012	4	Carboplatin Etoposide	2480 2490
013	1	Cisplatin Etoposide	147 588
014	1	Cisplatin Etoposide	101 600
015	4	Cisplatin Etoposide	399 1995
016	2	Carboplatin Etoposide	980 777
017	4	Carboplatin Cisplatin Etoposide	572 254 1187
018	2	Cisplatin Etoposide	317 1275
019	1	Carboplatin Etoposide	653 501
020	2	Carboplatin Etoposide	1422 1086
023	6	Cisplatin Etoposide	761 4572
024	4	Carboplatin Etoposide	3600 2349

**Table 3:** Last prior chemotherapy dose continued on study with amuvatinib

Subject	Agents	Nominal dose	Total dose (mg)
001	Carboplatin Etoposide	6 (target AUC) 80 (mg/m^2^)×3	660 420
002	Carboplatin Etoposide	5 (target AUC) 100 (mg/m^2^)×3	520 471
003	Carboplatin Etoposide	5 (target AUC) 100 (mg/m^2^)×3	652 201
004	Carboplatin Etoposide	5 (target AUC) 100 (mg/m^2^)×3	473 576
005	Carboplatin Etoposide	5 (target AUC) 100 (mg/m^2^)×3	619 606
006	Carboplatin Etoposide	6 (target AUC) 100 (mg/m^2^)×3	570 570
007	Carboplatin Etoposide	6 (target AUC) 80 (mg/m^2^)×3	640 477
008	Carboplatin Etoposide	5 (target AUC) 100 (mg/m^2^)×3	640 600
009	Carboplatin Etoposide	5 (target AUC) 100 (mg/m^2^)×3	672 555
010	Cisplatin Etoposide	100 (mg/m^2^)×3	152.25 480
011	Carboplatin Etoposide	3.75 (target AUC) 45 (mg/m^2^)×1	318 70
012	Carboplatin Etoposide	5 (target AUC) 100 (mg/m^2^)×3	620 630
013	Cisplatin Etoposide	100 (mg/m^2^)×3	147 588
014	Cisplatin Etoposide	120 (mg/m^2^)×3	101 600
015	Cisplatin Etoposide	100 (mg/m^2^)×3	100 501
016	Carboplatin Etoposide	6 (target AUC) 80 (mg/m^2^)×3	490 387
017	Cisplatin Etoposide	100 (mg/m^2^)×2	84 336
018	Cisplatin Etoposide	100 (mg/m^2^)×3	158 630
019	Carboplatin Etoposide	5 (target AUC) 100 (mg/m^2^)×3	653 501
020	Carboplatin Etoposide	6 (target AUC)100 (mg/m^2^)×3	737 540
023	Cisplatin Etoposide	120 (mg/m^2^)×3	127 765
024	Carboplatin Etoposide	6 (target AUC) 100 (mg/m^2^)×3	900 585

### Best response to treatment

**Figure 1:**
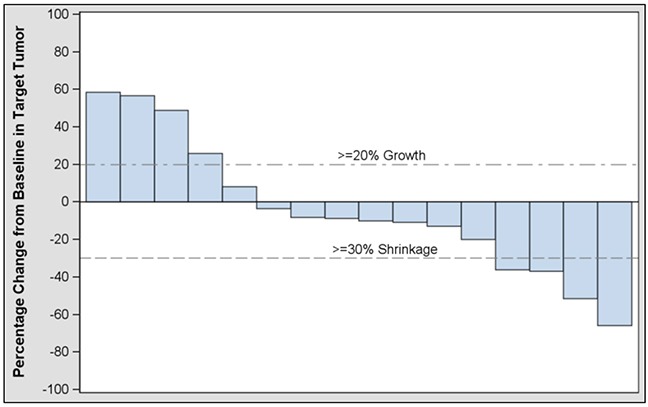
Investigator assessment of best change from baseline in target tumor in modified intent-to-treat subjects

Table 4 summarizes best response to treatment. Overall objective response rate (ORR) based on investigator assessment was four partial responses (PR) (17.4%) among the 23 subjects treated (95% CI: 5.0, 38.8). The primary endpoint of confirmed response by central assessment in evaluable subjects was 2/22 or 9% (95% CI: 1.1, 29.2); these two subjects were among the four who had a PR by investigator assessment. Figure 1 shows best change from baseline in subjects’ target tumor.

**Table 4:** Summary of best response to treatment (N = 23)

Disease control	RECIST v1.1 by investigator assessment(n)	ConfirmedRECIST v1.1 by central radiology (n)	Duration of disease control, range (days)
**CR**	0	0	--
**PR**	4^a^	2^a^	21-151^b^
**SD**	7	3^c^	81-256
**PD**	5	NA	NA
**NE**	7	NA	NA

CR = complete response; PR = partial response; SD = stable disease; PD = progressive disease; NE = not evaluable; NA = not applicable (progression was not required to be confirmed by central radiology assessment).

^a^Two subjects (001 and 023) had a confirmed PR in at least two assessments that was confirmed by independent radiologic review. Two remaining subjects (002 and 004) did not meet the primary study endpoint of objective response (RECIST v1.1 criteria).

^b^Calculation based on investigator assessment (n=4).

^c^Three subjects (008, 009, 012) had confirmed SDs in at least two assessments, but no independent radiologic review was performed.

#### Confirmed responders

**Figure 2:**
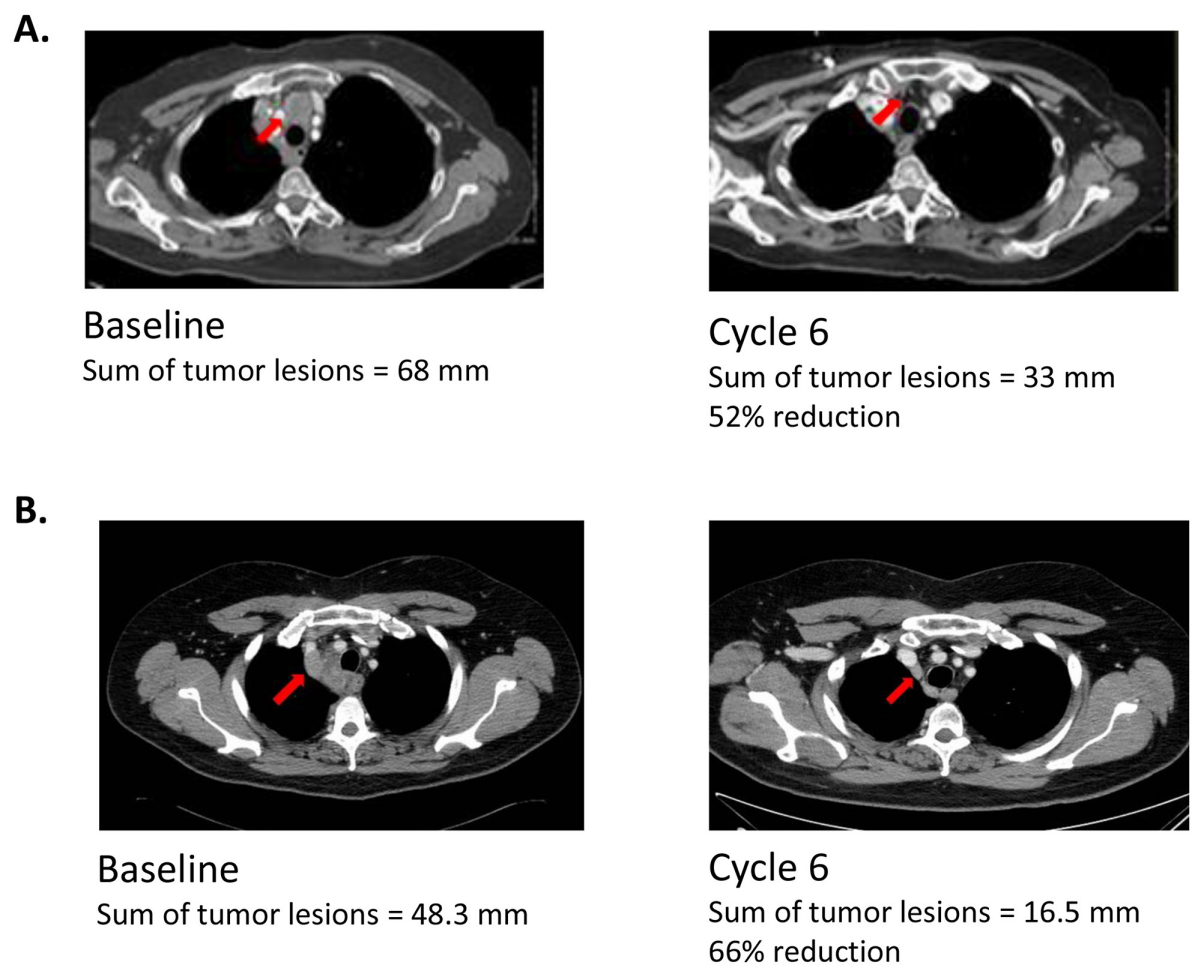
CT scans of confirmed responders Tumors indicated by arrows. **(A)** Subject 001 (67-yr-old female) Subject 001 entered the study with extensive-stage disease after previously receiving six cycles of prior chemotherapy with carboplatin/etoposide over a 15-week period (total dose, carboplatin 3746 mg; etoposide 2964 mg). Her best response to prior therapy was SD of unknown duration. She also previously received two doses of palliative radiation therapy: 4500 rad to the right lung, and 3000 rad to the brain. After enrollment into the study, she received six cycles of study drug over an 18-week period, along with continuation of her last prior EP cycle (total dose, carboplatin, 660 mg, and etoposide, 420 mg) before beginning amuvatinib and had a best response of PR (119 days) in at least two assessments confirmed by independent radiologic review. **(B)** Subject 023 (52-yr-old male) Subject 023 entered the study with extensive-stage disease after previously receiving six cycles of cisplatin/etoposide over a 16-week period with a best response of SD of four months’ duration. He also previously received two doses of palliative radiation therapy: 7500 rad to the right hilar mass and mediastinal lymph nodes, and 2500 rad to the right supraclavicular area. After enrollment into the study, he received six cycles of study drug over a 19-week period along with continuation of his last prior EP cycle (total dose, cisplatin 127 mg, and etoposide, 765 mg) before beginning amuvatinib and had a PR (151 days) in at least two assessments confirmed by independent radiologic review.

Subject 001, a 67-yr-old female who had a best response of stable disease (duration unknown) with prior carboplatin/etoposide, had a confirmed PR of 119 days in this study. At baseline, her sum of tumor lesions measured 68.0 mm, and after Cycle 6 a 52% decrease in tumor lesion sum to 33.0 mm was observed (Figure 2A).

Subject 023, a 52-yr-old male who had a best response of stable disease (4 months) with prior cisplatin/etoposide, had a confirmed PR of 151 days (4.96 months) in this study. At baseline, his sum of tumor lesions was 48.3 mm, and after Cycle 6 a 66% decrease to 16.5 mm was observed (Figure 2B).

#### Unconfirmed responders

Subject 002, a 44-year-old female whose best response to prior treatment is unknown, had a PR (by investigator assessment) of 21 days after Cycle 2; at baseline, her sum of tumor lesions measured 149.0 mm, and after Cycle 2 a 36.2% decrease in tumor lesion sum to 95.0 mm was observed.

Subject 004, a 74-year-old male who had a PR of 7 weeks with prior cisplatin/etoposide, had a PR (by investigator assessment) of 40 days (5.71 weeks) after Cycle 2; at baseline his sum of tumor lesions measured 38.0 mm, and after Cycle 2 a 31.6% decrease in tumor lesion sum to 26.0 mm was observed.

### Progression-free survival and overall survival

**Figure 3:**
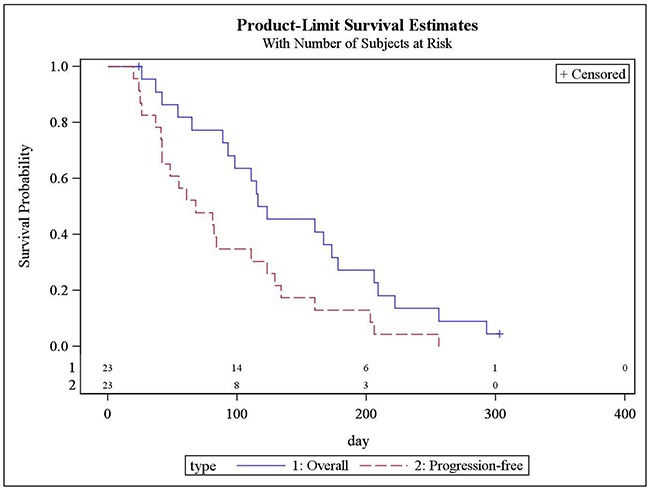
Progression-free survival and overall survival (Kaplan-Meier): N = 23

Progression-free survival (PFS) and overall survival (OS) are shown in Figure 3. Median PFS was 68 days (95% CI: 42,111), and median OS was 119 days (95% CI: 89,178).

### Disease control rate and duration of response

Disease control rate, based on investigator assessment, was 30.4% (95% CI: 13.2, 52.9). The rate was calculated based on 0 subjects with a complete response (CR), 4 subjects with a PR, and 3 subjects with confirmed stable disease (SD) ≥4 cycles (confirmed in at least two assessments but no independent radiologic review performed), from all subjects who received study treatment (*N* = 23). The median time to response per RECIST v1.1 for subjects with a PR (*N* = 4) was 49.5 days (range, 42-87 days).

Median duration of response per RECIST v1.1, based on investigator assessment, was 79.5 days (range, 21-151 days) for subjects with a PR (*N* = 4) and was 129.0 days (range, 81-256 days) for the subjects with SD (*N* = 7).

### Analysis of archival tissue for c-Kit and RAD51 expression

#### c-Kit expression in baseline tumor tissue

**Figure 4:**
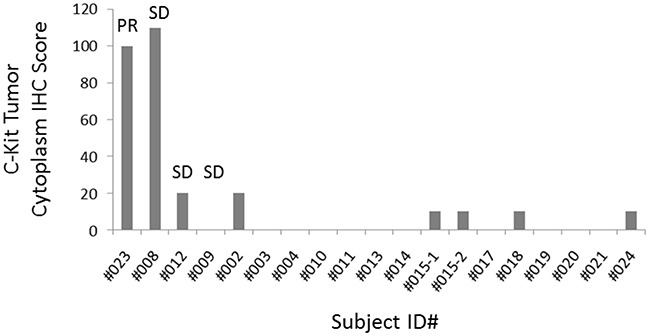
Baseline cytoplasmic c-Kit tumor IHC score c-Kit staining was quantified by using a 4-value intensity score (none = 0; weak = 1+; moderate = 2+; strong = 3+) and the percentage (0%-100%) of the extent of reactivity. A final score was obtained by multiplying the intensity and reactivity extent values (range, 0-300). Subject 023 (PR of 151 days) and subject 008 (SD of 256 days) showed the highest H-score. (Archival FFPE tissue was available in 18 subjects. c-Kit was not detected by IHC in 11 subjects).

Archival FFPE tissue was available in 18 subjects for testing of c-Kit expression by immunohistochemistry (IHC). c-Kit staining (Figure 4) was present at high levels (H-score ≥100) in subject 008 (H-score 110/300, SD of 256 days) and subject 023 (H-score 100/300, PR of 151 days). Conversely, c-kit staining was present at low levels (score range, 10-20) in 5 subjects. c-Kit was not detected by IHC in the remaining 11 subjects.

### RAD51 immunohistochemistry of baseline tumor tissue

**Figure 5:**
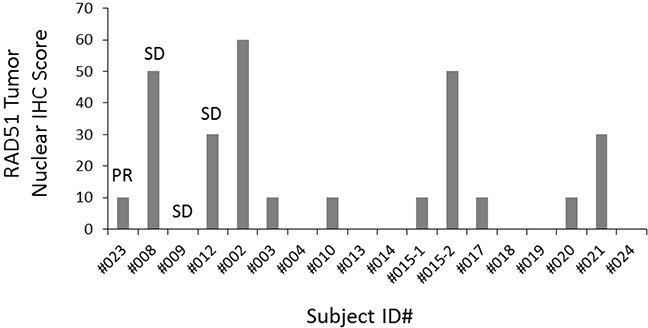
Baseline RAD51 tumor nuclear score RAD51 nuclear expression was quantified using a 4-value intensity score (0 = none; 1 = weak; 2 = moderate; 3 = strong) and the percentage (0%-100%) of the extent of reactivity. A final expression score was obtained by multiplying the intensity and reactivity extension values (range, 0-300).

Archival FFPE tissue was available in 17 subjects for testing of RAD51 expression by IHC. RAD51 staining (Figure 5) was present at high levels (score ≥100) in 0 subjects and was present at low levels (score range, 10-20) in 11 subjects. In 6 subjects, RAD51 was not detected by IHC. Subject 008 (who had high c-Kit expression and a clinical benefit from the combination of amuvatinib + EP) had a RAD51 score of 50, which was among the highest. Beyond this observation, no association was apparent between baseline RAD51 and response to amuvatinib + EP.

### Safety analyses

Amuvatinib 300 mg TID combined with EP chemotherapy was generally well tolerated and did not appear to add to the expected toxicity of the EP combination. Five subjects had AEs that led to death <30 days after receiving the last dose of study treatment. These AEs included failure to thrive, respiratory failure, and chronic obstructive pulmonary disease, and were all considered not related to study treatment.

Table 5 lists treatment-emergent AEs reported with the highest incidence (≥10%) in the study. The most common Grade 3 and 4 AEs among the 23 treated subjects were neutropenia (11/23, 48%), thrombocytopenia (9/23, 39%), anemia (5/23, 22%), fatigue (4/23, 17%), and leukopenia (4/23, 17%).

**Table 5:** Treatment-emergent adverse events in study subjects (N=23)

Preferred term	N (%)	Grade 3 and 4, n (%)
Thrombocytopenia	15 (65)	9 (39)
Neutropenia	14 (61)	11 (48)
Anemia	11 (48)	5 (22)
Fatigue	12 (52)	4 (17)
Diarrhea	9 (39)	1 (4)
Leukopenia	9 (39)	4 (17)
Nausea	9 (39)	0 (0)
Hypomagnesemia	9 (39)	0 (0)
Vomiting	7 (30)	0 (0)
Hyperglycemia	7 (30)	2 (9)
Hyponatremia	6 (26)	3 (4)
Hypokalemia	5 (21)	3 (4)
Decreased appetite	5 (21)	0 (0)
Dehydration	5 (21)	2 (9)
Dizziness	4 (17)	0 (0)
Peripheral sensory neuropathy	4 (17)	0 (0)
Dysgeusia	3 (13)	0 (0)
Hypocalcemia	3 (13)	1 (4)
QT prolongation	3 (13)	0 (0)
Edema peripheral	3 (13)	0 (0)
Urinary tract infection	3 (13)	0 (0)
Muscular weakness	3 (13)	0 (0)
Dysphagia	3 (13)	0 (0)
Hematuria	3 (13)	0 (0)
Respiratory failure	3 (13)	0 (0)
Cough	5 (21)	0 (0)
Dyspnea	5 (21)	1 (4)

## DISCUSSION

The primary objective of this study was to determine the effect of amuvatinib in combination with EP on overall objective response rate in subjects with platinum refractory SCLC. Although amuvatinib in combination with EP showed an ORR of 17.4% (PR by investigator assessment) and a disease control rate of 30.4% in this population of patients with SCLC, the regimen failed to meet the pre-specified efficacy threshold to justify further evaluation.

Our observed ORR was greater than that observed previously in patients with SCLC who were administered topotecan, the only drug currently approved for second-line treatment of SCLC. Ardizzoni et al [6] observed an ORR of 6.4% with topotecan administered as a 30-min infusion at 1.5 mg/m^2^/dose for five consecutive days in patients with refractory SCLC. Similarly, Perez-Soler et al [15] observed partial remissions in 3/32 etoposide-refractory patients (11%) administered topotecan at 1.25 mg/m^2^/dose over 30 min for five days every 21 days. In addition, a meta-analysis of results of clinical trials of investigational agents in relapsed SCLC showed an ORR of 17.9%, with a higher response rate of 27.7% (range, 0%-77%) for sensitive SCLC versus 14.8% (range, 0%-70%) for refractory patients; *P* = 0.0001 [16].

The safety profile of amuvatinib is consistent with previously reported results of manageable toxicity [14], and results from the current study provide additional support for the safety and tolerability of amuvatinib in combination with EP chemotherapy.

Nonclinical studies have shown RAD51 to be a potential predictor of tumor resistance in SCLC treatment [10]. Previous human studies have shown decreased RAD51 expression in skin biopsies from patients with advanced solid tumors who were treated with single-agent amuvatinib [13] as well as with amuvatinib in combination with standard therapy regimens [14]. However, we did not observe in the current study an association between baseline RAD51 levels and response to amuvatinib + EP. This may be due to the limited number of samples available for examination as well as the small number of patients who experienced clinical benefit in terms of objective response and durable control of disease.

The highest c-Kit scores were observed in two subjects with durable disease control to the combination of amuvatinib + EP: one with a PR of 151 days, the other with SD of 256 days. Of the 18 evaluable patients, 2/2 with high c-Kit expression had durable disease control, whereas 2/16 patients with low c-Kit expression had similar responses to treatment. The limited number of subjects with available tissue and positive clinical outcome, as well as the single-arm study design, made it impossible to establish a true association between c-Kit expression and benefit of amuvatinib in combination with EP. However, previous studies have identified higher c-Kit expression in SCLC cell lines when compared with non-small cell lung cancer [2]. Furthermore, overexpression and activation of c-Kit in SCLC surgical specimens was demonstrated to be prognostic in SCLC [17–19]. Micke et al [17] showed that patients with c-Kit negative extensive stage SCLC have higher median survival rates and negligible response to old-generation chemotherapy (eg, cyclophosphamide and adriamycin combination, or single-agent cyclophosphamide, adriamycin, or etoposide). Previous efforts to target c-Kit for therapeutic benefit in c-Kit expressing SCLC have been unsuccessful: in a single arm Phase 2 study, Schneider et al. [20] enrolled 14 patients with c-Kit expressing tumors, of which eight patients with a PR to irinotecan/cisplatin received imatinib maintenance. No objective responses to imatinib were observed, although three patients achieved SD of 12-25 weeks with median PFS and OS respectively of 4.3 months (95% CI, 2.9-4.8 months) and 7.8 months (95% CI, 5.7-10.0 months). Similarly, Dy et al [21] reported results of a study (Simon 2-Stage design [22]) of imatinib in patients with recurrent, refractory c-Kit-expressing SCLC. The study was terminated early following an interim analysis showing lack of efficacy. As amuvatinib is a multikinase inhibitor with activity against c-Kit and potentially other relevant SCLC targets in preclinical studies, it is tempting to ascribe the durable clinical benefit to amuvatinib + EP observed in our study in part to the high c-Kit scores obtained in those two subjects.

While future studies of amuvatinib in combination with DNA-damaging agents may be justified given the safety profile and nominal efficacy signal we observed, thoughtful patient selection will be important to ensure future studies are successful in demonstrating the clinical impact in SCLC and other cancer types.

## MATERIALS AND METHODS

### Study agent and regulatory information

Amuvatinib hydrochloride (C_23_H_22_ClN_5_O_3_S) is a fully synthetic carbothioamide that was administered in this study as lipid-suspension capsules containing drug product and excipients.

Seven study centers in the United States and one in Poland participated in the study. The study protocol was reviewed and approved by the governing institutional review boards (or ethics committees) overseeing research in human subjects at each center. Participating subjects provided written informed consent.

### Subject population

Eligible subjects were males or females ≥18 years of age with histologically or cytologically confirmed SCLC and measurable SCLS according to the Response Evaluation Criteria in Solid Tumors (RECIST version 1.1) that met one of the following criteria: disease progression by RECIST at any time during initial or rechallenge with EP chemotherapy; relapse by RECIST within 90 days after completing EP chemotherapy; SD by RECIST as best response after at least two ≥21-day cycles of EP chemotherapy.

Subjects were required to have Eastern Cooperative Oncology Group (ECOG) performance status 0 to 2, adequate bone marrow (absolute neutrophil count [ANC] ≥1,500/mL, platelet count ≥100,000 IU/L), renal function (serum creatinine <1.5 × upper limit of normal [ULN]), hepatic function (aspartate aminotransferase and alanine aminotransferase <2.5 × ULN, total bilirubin <1.5 × ULN), pulmonary function (O_2_ saturation >90% in room air), and cardiac function (no clinically significant ECG findings and left ventricular ejection fraction >50%). Subjects were required to have a measurable QTc interval (by Bazett's or Fridericia's formula) of <450 msec by 12-lead ECG and were also required to have electrolytes (magnesium, calcium, and potassium) within the normal limits of the institution at screening or corrected before the first day of amuvatinib dosing. Subjects could not have history of arrhythmias, angina pectoris, risk factors for Torsades de Pointes, ECG evidence of a myocardial infarction, or any Class 3 or 4 cardiac disease as defined by the New York Heart Association Functional Classification. Female subjects of childbearing potential could not be pregnant or breastfeeding and must have had a negative pregnancy test at screening.

Subjects excluded from participating were those who had previous exposure to amuvatinib; who came off prior chemotherapy due to toxicity; who have ongoing toxicity from prior treatment; who have mixed SCLC and non-small cell lung cancer, or large cell lung cancer. Subjects were excluded who had untreated, unstable, or symptomatic brain metastasis; history of a different malignancy within the last three years other than adequately treated and completely excised cervical cancer in situ, ductal carcinoma in situ, basal cell or squamous cell carcinoma of the skin. Subjects were also excluded if they had hypersensitivity to amuvatinib components of the drug product or any other agent associated with the study, were a poor medical risk, or had a life-threatening illness or other medical condition that might interfere with study outcomes. Patients must not have been treated with an investigational drug within three weeks of the first dose of study drug.

### Study design

This was a Phase 2, multi-center, open-label, single-arm study of amuvatinib in combination with EP in subjects with extensive-stage or limited-stage SCLC who had not responded to standard treatment or who had relapsed after standard treatment (within 90 days of completing EP). The primary objective of the study was to assess overall objective response rate (complete response [CR] or partial response [PR]) to amuvatinib in combination with EP chemotherapy) per RECIST v1.1.

The primary study endpoint was overall objective response rate (CR or PR per RECIST v1.1); secondary endpoints were progression-free survival (PFS), overall survival (OS), disease control rate, duration of response, incidence and severity of adverse events (AEs), and assessment of exploratory biomarkers in selected subjects.

This study followed an optimal Simon 2-stage design [22] using the following parameters: α=10%, β=10%, p_0_=10% and p_1_=25%. Based on these parameters, three of 21 evaluable patients in Stage 1 were required to achieve confirmed objective response for the study to proceed to Stage 2 (accrual of additional 29 patients for a total sample size of 50 subjects).

### Study procedures

#### Dosing

Subjects were administered 10 × 30 mg amuvatinib (300 mg total) lipid-suspension-filled capsules orally three times daily (TID) continually for 21 days (21-day cycles), taken in a seated position over a 30-minute period with water and, if feasible, with food. A 3-day amuvatinib run-in period (±1 day) occurred in Cycle 1 prior to the 21-day treatment period.

The EP chemotherapy consisted of cisplatin or carboplatin, and etoposide (VP-16). Subjects received the same EP chemotherapy regimen (dose and schedule) as that on which they progressed or relapsed before study entry. Dosing started on Day 1, Cycle 1, after the three-day run-in period of amuvatinib (±1 day) described above. Study treatment with chemotherapy and amuvatinib continued as long as the subject benefitted and no unacceptable toxicity occurred. No comparative treatment or placebo was used in this study.

Dose escalation was not allowed at any time during the study. Dose delay and/or adjustment of EP followed standard practice at the study center.

### Supportive care

Appropriate hydration and supportive care (eg, antiemetics, antibiotics, or other growth factors) were permitted according to study center standards. Corticosteroids were permitted if the dose had been stable for at least two weeks before study entry. Medications with dysrhythmic potential were not recommended, and medications with the potential for QT prolongation were to be used with caution.

### Exploratory biomarkers assessment

#### c-Kit

c-Kit is a target of amuvatinib and has been previously shown to be overexpressed in a subset of SCLC [23]. Therefore, we investigated whether tumor c-Kit expression levels corresponded with response in this study. To assay baseline c-Kit expression, formalin-fixed paraffin-embedded (FFPE) sections from pre-treatment tumor biopsies (or archival samples from before study entry) were deparaffinized and rehydrated by heating overnight at 56°C, incubating 3×5 minutes in xylene, followed by three-minute washes in 100%, 95%, 70%, and 50% ethanol, followed by two washes for five minutes in deionized water. Antigen retrieval was achieved by bringing slides to a boil in 10 mM sodium citrate buffer (pH 9.0), maintaining a sub-boil for 30 minutes, and cooling at room temperature for 20 minutes. Endogenous peroxidase activity was quenched by incubating sections in 3% H_2_O_2_/10% methanol for 15 minutes. Sections were blocked for one hour in blocking solution, and 5% normal goat serum was diluted TBS-T (Tris Buffered Saline with Tween 20) at room temperature. Anti c-Kit primary antibody (A4502, DAKO, Carpenteria, CA) was diluted 1:400 in blocking solution and incubated for 1 hour at room temperature. Sections were washed three times in TBS-T before adding 1-3 drops Envision Dual Link+ (DAKO) and incubating in a humidified chamber for 30 minutes at room temperature. Staining was developed using Signal Stain DAB (Cell Signaling Technology, Danvers, MA) chromogen before counterstaining with hematoxylin. Sections were finally dehydrated by washing twice in 95% ethanol, 100% ethanol and xylene, and then mounting coverslips with Permount (Fisher, Pittsburgh, PA). c-Kit staining (H-score) was quantified by using a four-value intensity score (0, none; 1+, weak; 2+, moderate; 3+, strong) and the percentage (0%-100%) of the extent of reactivity. A final score was obtained by multiplying the intensity and reactivity extent values (range 0-300) [24]. A c-Kit score of ≥100 was considered high (ie, relatively higher-expressing and associated with more favorable clinical benefit), and ≤20 was considered low.

### RAD51 immunohistochemistry of baseline tumor tissue

Proteomic analysis of preclinical SCLC models has previously shown overexpression of several DNA repair proteins in SCLC compared with non-small cell lung cancer (NSCLC) [2, 25]. Therefore, because the predicted mechanism of action of amuvatinib in platinum-refractory SCLC was downregulation of RAD51 leading to sensitization to DNA-damaging agents (in this case, EP chemotherapy), we investigated whether baseline RAD51 levels were associated with patient responses. Immunohistochemical staining for RAD51 was performed on whole-section samples. Briefly, 4-μm FFPE tissue sections were deparaffined, hydrated, and processed in Leica BOND-MAX (Leica Microsystems Inc., Buffalo Grove, IL). Slides were incubated with the primary antibody (RAD51 (prediluted, Abcam, Cambridge, MA) 1:2). The antigen retrieval was performed with Leica Bond retrieval solution; the IHC reaction was performed according to standard protocol from Leica Bond, including 20 min of protein blocking, counterstained with hematoxylin, then dehydrated and mounted. RAD51 nuclear expression (H-score) was quantified as for c-Kit [24].

### Statistical methods

All subjects who received any study treatment were included in the statistical analyses. Demographic and baseline characteristics were summarized using descriptive statistics.

Subject-reported and investigator-observed AEs were coded using the Medical Dictionary for regulatory Activities (MedDRA) version 14.0. AE severity was graded according to National Cancer Institute - Common Terminology Criteria for Adverse Events (CTCAE). AE incidence was summarized by MedDRA system organ class and preferred term, CTCAE grade, and relationship to study treatment.

Efficacy was assessed by the following:

- Objective response rate (ORR), defined as CR or PR according to RECIST v1.1 in two assessments at least four weeks apart and confirmed by independent radiologic review.
- Progression-free survival (PFS), defined as the number of days from the day the subject received the first dose of amuvatinib to the date of documented disease progression by RECIST v1.1 or death, whichever occurred first.
- Overall survival (OS), defined as the number of days from the day the subject received the first dose of amuvatinib to the date of death.
- Disease control rate, defined as the percentage of subjects who achieve a response of CR, PR, or SD ≥4 cycles.
- Duration of response for CR or PR was determined from the earliest assessment of CR or PR until disease progression or death or last contact date, whichever occurred first. Duration of response for SD was computed from the day study treatment was first taken to the date of disease progression or death or last contact date, whichever occurred first.

ORR was summarized by number and percent of subjects with a CR or PR along with the 95% confidence interval (CI) based on a binomial distribution. Time to response was summarized for centrally confirmed responders (CR and PR) using mean, standard deviation, minimum, median, and maximum. Response rates and disease control rate were estimated along with the 95% CIs based on binomial distributions.

Time-to-event endpoints were summarized using Kaplan-Meier analyses. PFS for subjects who withdrew from the study without documented disease progression (by RECIST v1.1) were censored on the day of withdrawal. Survival for a subject who was lost to follow-up was censored on the last date the subject could be confirmed living.
